# Only females in poor condition display a clear preference and prefer males with an average badge

**DOI:** 10.1186/1471-2148-10-261

**Published:** 2010-08-27

**Authors:** Matteo Griggio, Herbert Hoi

**Affiliations:** 1 Konrad Lorenz Institute for Ethology, Savoyenstrasse 1 a, A-1160, Vienna, Austria

## Abstract

**Background:**

Female condition-dependent variation in mate preference may have important evolutionary implications, not only within the same population but also among populations. There are few experiments, however, on how condition and/or genotype influences female mate preferences. The black throat patch of the male house sparrow, *Passer domesticus*, is an intensively studied plumage trait. It is often referred to as a 'badge of status' and seems to be involved in female mate choice, but differences exist among populations. Between-population variation in mate preference may occur for condition-dependent mate preferences. We tested the hypothesis that female preference may vary with female quality (body condition). Therefore, we measured female preference for badge size using an aviary two-choice test in which females were presented with two males that had different sizes of badges (enlarged or averaged).

**Results:**

Overall we did not find a female preference for enlarged or average badges, but low-quality females spent more time near average badge males. Conversely, high-quality females did not show a clear preference.

**Conclusions:**

Collectively, these results indicate that female preference varies with female quality. Differences in female condition are causes of within-population variation in mating preferences. To our knowledge, our results provide one of the first experimental evidences that variation in preference for a male ornament is associated with female condition. In our study, however, only females of low condition displayed a clear mate preference. Differences observed among populations could be partly explained by differences in female condition.

## Background

Mate choice is one important force driving sexual selection [1,2]. Usually females select males on the basis of vocalisations, behavioural displays, pheromones and morphological traits which may indicate the quality of the males [3]. Many studies on sexual selection focus on the signalling function and fitness consequences when females mate with males that exhibit particular male ornaments [3]. There is little experimental evidence on how intrinsic quality of the choosing individual is integrated in the mate-choice process and affects preference decisions [4,5]. Genetic compatibility may be responsible; the best male varies between different female phenotypes and in its extreme form every female may have its own 'best' male [6]. Alternatively, mate preference may depend on the direct costs and benefits of mating with certain males, which may vary among females. Several models of state-dependent mate choice (for a review see [7]) predict that high-quality females are more choosy and invest more in mate sampling, assuming that mate preference should be more beneficial for high-quality females and low-quality females have less to gain from discrimination. Low-quality females are expected to be less choosy or spend less time in mate sampling because they are less successful in attracting a mate or are less successful in mate competition with other females [8,9].

For bearded tits, Hoi [10] demonstrated that only a dominant, high-quality, female initiates males to chase her and she copulates with the 'best' male, which is not necessarily her social partner. Females in poor condition try to avoid this energetically costly tactic because every male, even of low quality, would overcome the female's resistance. Consequently, low-quality females would not benefit by initiating male competition to chase them. Another example in line with the predictions of these state-dependent models is female sticklebacks, *Gasterosteus aculeatus*. They choose between males according to the intensity of red coloration, which decreases as males become more parasitised [11]. Females prefer redder males, but this varies with the viability of the female. Indeed, females in better condition prefer males that are red, whereas females in poorer rearing condition prefer males that are orange [4]. Several other studies found evidence for condition-dependent variation in mate choice. In *Photinus ignites *fireflies, laboratory-fed females showed lower overall responsiveness for male courtship flashes relative to control females that did not feed in the laboratory [5]. Female zebra finches, *Taeniopygia guttata castanotis*, spent less time associating with attractive males when their own physical condition was altered by clipped wing feathers [12]. Experimentally infected female wild turkeys, *Meleagris gallopavo*, assessed a larger set of males, probably to increase the opportunity to obtain complementary parasite-resistance genes [13]. Our knowledge of the effects of female condition, or phenotypic quality, on mate preference is limited.

The house sparrow, *Passer domesticus*, is a model organism for studying sexual selection in relation to plumage variation (for a review see [14]). The melanin-based black throat patch is an honest indicator of condition [15-20] and is often referred to as a 'badge of status' (intrasexual selection). Males with large badges acquire territories of a better quality than small-badged male sparrows and reproductive success is better for females paired with large-badged males because their territories include safe nest sites. Moreover, large-badged males are more efficient mate guarders than small-badged males ([17] and references therein) and reproductive success increase with badge size [21]. An experimental study demonstrated a relationship between food quality and the subsequent expression of the badge [20]. The badge size seems to be involved in female mate choice, but differences exist among populations (see Discussion). Moreover, in some populations it seems that males with small- or average-sized badges invested more in parental care than other males (see Discussion). All these differences among populations could be partially owed to differences in female preferences (for a review see [22]). The objective of this study was to examine how female condition influences female mate preference based on badge size in house sparrows. In particular we tested two predictions. The first prediction is that females prefer males with larger badges. The second prediction is that high-quality females are more discriminating than females in poor condition. The individual body condition was calculated by dividing body mass by (tarsus length)^3 ^. This measure of body condition reflects the relative weight at a given size. Previous studies showed that this measure is negatively correlated with a stressful growth situation, in terms of both nutritional and hormonal stress, in passerine birds (e.g. [23-25]). In house sparrows, female condition is a positive predictor of laying date, clutch size and number of fledglings ([26] and see Methods). We measured female preferences for male badge size using an aviary two-choice test in which females were presented with two males whose badge was experimentally enlarged to the average size or to the maximum size observed in the natural range.

## Results

**Figure 1:**
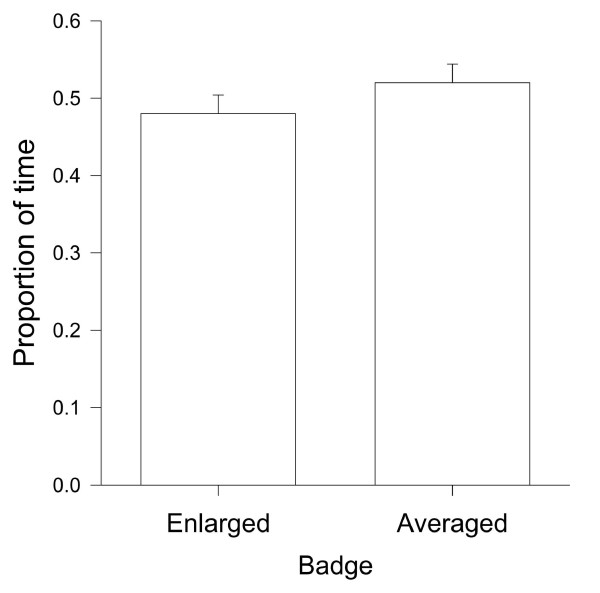
**Proportion of time females spent near the window of each male cage (Enlarged or Averaged badge males) for all females combined (N = 85)**. Given are means + s.e.

**Figure 2:**
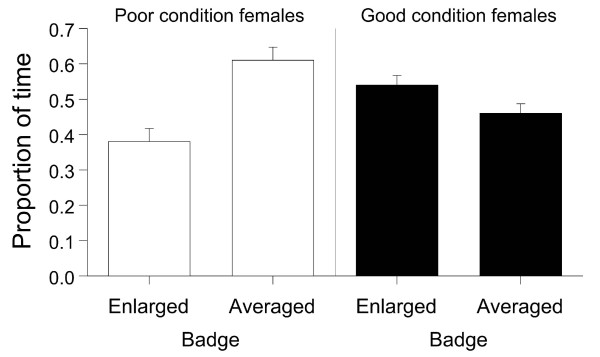
**Mean time + s.e. females spent near the window of each male cage (Enlarged or Averaged badge males) separated for low (open bars, N = 35) and high quality females (filled bars, N = 50)**.

**Figure 3:**
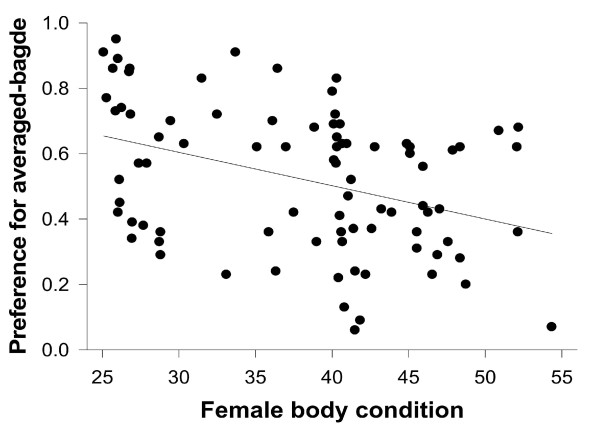
**Relationship between female preference for the smaller badged male (Averaged badge male) and her body condition**. Proportion is the time female spent near the window of each male (Enlarged or Averaged badge male) over the total time in the choice area.

Considering all females together, we did not detect any particular female preference, (paired t-test: t = 0.938, df = 84, P = 0.351; Figure 1). After dividing females according to their condition into two groups, however, we found that low-quality females preferred average-badged males (paired t-test: t = 3.072, df = 34, P = 0.004; Figure 2). On the other hand, high-quality females did not show a clear preference (paired t-test: t = 1.523, df = 49, P = 0.134; Figure 2). Therefore, we examined the correlation between females' preference and their own condition. We found that overall there was a strong negative correlation between the proportion of trial time females spent investigating average-badged males in relation to the condition of the female (Pearson correlation test: r = 0.379, P < 0.001, N = 85; Figure 3). In the generalised linear mixed models, there were no effects of morphological traits and age, for both males and females, on female preferences (see Table 1). On the other hand, there was a significant effect of male group (average or enlarged badge) on female preferences, and the effect of the interaction between female groups (high or low condition) and male groups on female preferences was also significant (see Table 1). Furthermore, when we excluded female groups from the model the significant effect of male manipulation (male group) on female preference disappeared (male group: F _1, 159 _= 1.915, P = 0.168), indicating again that female preference for enlarged or average black bibs is not evident when all the females are considered together. Lastly, there was no correlation between the female body condition and the difference (more preferred minus less preferred male) in the proportion of time females spent in front of the two males (Pearson correlation test: females altogether, r = -0.185, P = 0.09, N = 85). This indicates that females of different condition did not display a different degree of preference.

**Table 1:** Model summary of the Generalized linear mixed models testing for effects on female mate preferences.

Factor	*Full model*		*Minimal model*	
	
	*F_1,157_*	*p*	*F_1,166_*	*p*
Female group (body condition)	0.004	0.947	0.000	0.997
Male group (badge manipulated)	4.561	**0.024**	5.048	**0.026**
Female wing length	0.002	0.965		
Female weight	0.014	0.967		
Female tarsus length	0.019	0.891		
Female age	0.199	0.656		
Male wing length	0.040	0.842		
Male weight	1.712	0.193		
Male tarsus length	1.443	0.231		
Male age	2.192	0.141		
Male body condition	1.464	0.228		
Female group × Male group	22.797	**< 0.001**	23.734	**< 0.001**

Significant values are shown in bold.

## Discussion

The results of these experiments reveal (i) a lack of general female preference for the badge enlarged to the maximum size; (ii) selectivity in mate choice varies with female condition and low-quality females prefer males with smaller badges; (iii) in contrast to the prediction low-quality females are more discriminating. To our knowledge, our results provide one of the first experimental evidences that variation in preference for a male ornament is associated with female condition, in particular for females with poorer body condition. A recent study on zebra finches demonstrated that high-quality females preferred high-quality males' mate-advertising songs, whereas low-quality females preferred low-quality males' song [27]. Our results are partially in line with this previous work, but in our study only females in poor condition displayed a clear mate preference.

Cotton and co-workers [7] suggested that condition-dependent preference depends on female ability to pay the costs of or gain benefits from her discrimination. In particular, in their review they supported the hypothesis that high-quality females show the strongest mate preference. Our results seem to be contrary to their predictions; indeed low-quality females displayed a clear mate preference. These findings suggest that the relative importance of the benefits (genetic or parental care) varies with changing female condition and female house sparrows may adjust their preference for badge sizes accordingly (as reported for *Photinus ignitus *fireflies [5]). In a wild house sparrow population, males with average-sized badges invested more in parental care than other males [28], which is in line with our results. Therefore, poor-quality females may have more to gain directly from choosing average-sized badge males with high parenting ability. A female in poor condition, without the male parental contribution, would have fewer chances to breed successfully than a high-quality female that could even breed without paternal care. This has to do with the dual signalling function of the black melanin badge: contrasting male badge size may signal different male qualities. Consequently, whether to choose a male according to genetic quality (large badge) or paternal quality (small badge) may depend on intrinsic female quality. This is in line with Gowaty's 'female constraint hypothesis' [29-31]. According to this hypothesis, low-quality females should try to increase their fitness by selectively mating with a good father. High-quality females should be more selective when choosing the genetic father (e.g. seeking more likely extra-pair copulations). Females hence may trade parental quality (direct benefits) for genetic quality (indirect benefits). This may provide one explanation for genetic variance in sexually selected male signals.

An alternative explanation is that low-quality females are trying to avoid direct attacks from more ornamented males (see 'code' hypothesis [32]). Aggressive chases are a frequent part of sexual behaviour in house sparrows [33] and females may prefer a safe environment by mating with less aggressive males. Sexually experienced female Japanese quails, *Coturnix japonica*, that are offered a choice between two conspecific males previously observed engaging in an aggressive encounter prefer to affiliate with the less aggressive male [34]. Therefore, it seems that in Japanese quail, a species in which male courtship and mating are potentially harmful to females, females keep away from relatively aggressive males in order to avoid physical punishment.

Variation in female preference according to badge size of male house sparrows exists among all populations but the direction of the preference differs. In a Danish population males with larger badges obtained a mate earlier during the breeding season than males with smaller badges. Females displayed more copulation solicitations in front of male dummies with large badges [16]. Conversely, on Lundy, an island 20 km off the south-west coast of England, females preferred males with smaller badges and produced a higher number of offspring with such males [35,36]. Moreover, males that invested more in current reproduction subsequently developed a smaller badge. This indicates a trade-off between the reproductive effort and the expression of this sexual ornament. In our study population, males with smaller, but not the smallest, badges started to breed earliest, had the largest clutches and invested in parental care more than other males [28]. An experimental study in Spain did not find any support for reproductive success being based on badge size [37]. Lastly, in a controlled laboratory study, females showed no preference for large-badged males [38]. All these differences observed among populations could be partly explained by differences in female condition. Indeed it is not only female intrinsic quality but also environmental quality, e.g. food availability [39], which may influence female mate choice [29].

## Conclusions

When we examined female choice in the usual way house sparrows did not show a preference for males with larger badges. The preference, however, was highly variable between individuals. In terms of female quality, poor-quality females preferred smaller-badged males. Our results suggest that differences in female condition are the causes of within-population variation in mating preferences. Even differences observed among populations could be partly explained by differences in female condition. It would be interesting to know how environmental heterogeneity, female condition and the mate preference pressure they create influence the evolution of a sexually selected trait. Depending on the costs and benefits of parental investment the female condition-dependent preference for a sexually selected trait related to male parental investment may vary across populations.

## Methods

### Maintenance of captive birds and morphological measurements

The mate choice experiments were carried out at the Konrad Lorenz Institute for Ethology (Vienna, Austria) with a total of 96 males and 85 females at the start of the spring mating period. Males and females were collected during July and August at the Vienna Zoo (47°56'N, 16°45'E) and they were kept in nine large outdoor aviaries (3.5 m × 3.5 m × 3 m; about twenty individuals per aviary). Sexes were visually but not acoustically isolated throughout the study until the start of the experiment. All aviaries were equipped in the same way with vegetation, several perches (about seven per aviary) and nest boxes (eight per aviary). Commercial food for granivorous passerines and water were provided ad libitum. The start of breeding immediately after the experiment and the performance of several breeding attempts suggest that the housing conditions and experiment were appropriate and had no negative effect on the birds' health or condition. All procedures followed the Association for the Study of Animal Behaviour/Animal Behaviour Society Guidelines for the Use of Animals in Research and Austrian laws. Body mass (± 0.1 g), tarsus length (± 0.1 mm), and tail and wing length (± 1 mm) [34] were taken prior to the transfer into experimental chambers. Badge size was measured (with callipers, ± 0.1 mm) from the base of the bill to the point on the black breast at which the black feathers finished. This measurement was used instead of the area of the badge, because measurement of the width across the whole black area is less accurate [40]. In any case this measurement is the main determinant of overall badge size (authors' pers. obs., see also [36]).

Individual body condition was calculated by dividing body mass by (tarsus length)^3 ^owing to small values the body condition indices were multiplied by 10^4^. Similar results were obtained using the residual from a regression of body mass and tarsus length (r = 0.97, p < 0.0001, N = 30). This measure of body condition reflects the relative weight at a given size. In our field study population (Vienna Zoo) females in better condition laid larger clutches and started to breed earlier (laying date: r = 0.77 p < 0.0001; clutch size: r = 0.64 p < 0.001, N = 30). We divided our population into two groups of females according to female body condition and taking the average value as the separator between the two groups (range: 25.08-54.35, average: 38.01). Females in poor condition (high quality females: 43.91 ± 0.57 SE, N = 50) and females in good condition (low quality females: 29.59 ± 0.68 SE, N = 35; student *t *test: *t *= 16.13, *P *< 0.001).

### Badge manipulation and mate preference trials

Ninety-six male house sparrows were randomly divided in two groups (enlarged and average groups), and they were placed in indoor single cages (50 × 50 × 50 cm); seeds (commercial food for granivorous passerines) and water were available ad libitum before and during the experiments. We enlarged the badge using a black marker to cover the original badge and to enlarge it to a size at the limit of the natural variation (50 mm, see also [19]) in the case of the enlarged group, and to 35 mm in the case of the average group. Mean badge size, wing, tarsus, tail length and body mass (weight) and body condition of the two groups of males were not statistically different before manipulation (see Table 2).

**Table 2:** Phenotypic differences between the two groups of male house sparrows used for the experiment prior to the experiment.

Male trait	Averaged Group (N = 48)	Enlarged group (N = 48)	*F_1,94_*	*p*
Original badge size (mm)	30.29 ± 0.30	31.63 ± 0.80	2.474	0.119
Body mass (g)	26.51 ± 0.29	26.29 ± 0.27	1.117	0.293
Wing length (mm)	77.04 ± 0.04	77.48 ± 0.03	0.770	0.382
Tarsus length (mm)	19.81 ± 0.11	19.80 ± 0.15	0.006	0.938
Body condition	34.39 ± 0.83	34.24 ± 0.97	0.025	0.874

ANOVA test and mean ± SE are given.

We measured the proximity preference of 85 females (according to condition: 35 females of poor body condition and 50 females of good body condition) that were given a preference of an enlarged or an average male in a dichotomous choice chamber (2 × 0.5 × 0.5 m; for similar experimental apparatus see [41,42]). In each trial a different stimulus set of males was used. All birds were unfamiliar with each other because they came from different visually and acoustically separated aviaries. Males and females were allowed to acclimatize to their compartments for at least 30 min before observations started. After acclimatisation, the position of the female was recorded every 10 sec for 2 h. Behavioural observations were carried out from a hide placed approximately 4 m from the experimental apparatus. The mate preference trials were performed between 0800 and 1100 hours. Males with increased and average badges were alternated with respect to left and right compartments among consecutive trials to control for position effect. The female, placed in a central aviary, was allowed to choose between enlarged and average patched males placed individually in the two adjacent chambers. Opaque dividers were erected on the two sides of the central aviary to avoid visual interaction between the two males. The dividers also prevented the females from simultaneously observing the two males. Two perches were positioned so that the female could perch in the left or in the right side of her aviary. Perches had a line traced in the middle of their length, which corresponded to the edge of the separators. This line marked the part of the perch from which a female could observe the male in the nearby compartment. As a measure of proximity preference, we measured the time spent by a female on the perch in front of either male's compartment (choice time). Female mate preference was expressed as the proportion of time in front of each male over the total time in the choice area (e.g. [43-46]). When females perched in the zone where visual access to both male compartments was obscured (i.e. the neutral zone), we recorded no preference with respect to either male (neutral time). We defined the preferred male as the male with whom the female spent most of the time.

### Statistics

Data were analysed with generalised linear mixed models. The female mate preference was entered as the independent variable (after arcsine transformation). Fixed effects were female group (good or poor condition) and male group (average or enlarged badge). Wing, tarsus length, weight and age for both males and females were entered as covariates in the model. Female individual identity was included as a random factor to control for the dependent data. From the full model non-significant variables were sequentially removed in a backward stepwise procedure after the removal of the least significant term (*P *< 0.005). All results are presented as mean ± SE. All tests are two-tailed. We performed statistics using SPSS 17.0. Data were checked to ensure that they met the assumptions of parametric statistics. Female mate preference was also analysed with a paired *t *test on arcsine-transformed data.

## Authors' contributions

MG and HH designed the project. HH performed the experiment and MG analysed the data and wrote the paper.
